# Effects of SoundBite Bone Conduction Hearing Aids on Speech Recognition and Quality of Life in Patients with Single-Sided Deafness

**DOI:** 10.1155/2020/4106949

**Published:** 2020-09-08

**Authors:** Qiong Luo, Ying Shen, Ting Chen, Zhong Zheng, Haibo Shi, Yanmei Feng, Zhengnong Chen

**Affiliations:** ^1^Department of Otolaryngology Head and Neck Surgery, Shanghai Jiaotong University Affiliated Sixth People's Hospital, Shanghai Key Laboratory of Sleep Disordered Breathing, Shanghai, China; ^2^Department of Stomatology, Shanghai Jiaotong University Affiliated Sixth People's Hospital, China

## Abstract

**Objectives:**

To analyze the clinical application of SoundBite bone conduction hearing aids by assessing the improvement of speech recognition and the scores of the benefit scale questionnaire for patients with single-sided deafness (SSD).

**Design:**

Nine patients aged 24 to 61 years with SSD for more than 3 months were enrolled in this study. The patients could understand and repeat Mandarin and have good compliance with the study. The measurements were evaluated before and after one month of wearing hearing aids using the pure tone audiometry threshold, speech recognition in quiet and in noise, and the Glasgow Benefit Inventory (GBI) benefit scale score.

**Results:**

Pure tone audiometry results showed that the average hearing threshold of good ears and bad ears was 11.4 ± 2.6 dB HL and 89.9 ± 6.4 dB HL, respectively. The average hearing threshold of bad ears after wearing hearing aids was 23.5 ± 9.0 dB HL. Statistical analysis showed that the hearing improvement for the bad ears after wearing hearing aids was significant. The speech audiometry results showed that the disyllable word recognition score of the bad ears in quiet increased significantly at 50 dB SPL by 40 ± 12 percentage points and at 65 dB SPL by 71 ± 15 percentage points. As for the speech recognition in noise, when the signal sound came from the bad ear side and the noise from the good ear side (S_SSD_N_AH_), the speech recognition score (SRS) significantly increased by 17 ± 6 and 9 ± 4 at a signal-to-noise ratio (SNR) of -2 dB and -5 dB, respectively, after wearing the hearing aids. When the signal sound came from the front of the patient and the noise from the bad ear side (S_0_N_SSD_), the SRS scores were reduced by 5 ± 5 and 7 ± 5 percentage points at SNR equal to -2 dB and -5 dB, which was significantly different from that before wearing the hearing aids. When the signal and noise both came from the front of the patients (S_0_N_0_), the SRS was not significantly increased by 5 ± 4 percentage points at SNR equal to -2 dB compared to before wearing hearing aids. However, the SRS was significantly increased by 5 ± 2 percentage points at SNR equal to -5 dB compared to before wearing hearing aids. The average total GBI score was 31 ± 12 for the nine patients, with an average score of 32 ± 10, 31 ± 8, and 30 ± 7 for general conditions, social support, and physical health, respectively. The results of the questionnaires showed that patients' quality of life was improved after wearing SoundBite bone conduction hearing aids.

**Conclusions:**

SoundBite bone conduction hearing aids are a good choice for patients with SSD, as it could improve the speech recognition ability of patients both in a quiet and noisy environment and improves the quality of life after wearing hearing aids.

## 1. Introduction

Single-sided deafness (SSD) refers to severe to profound sensorineural hearing loss on one side (>70 dB HL) and an average hearing threshold of 0.5 to 4 kHz ≤25 dB HL on the good ear [1]. People with SSD generally do not wear hearing aids because they can depend on the good ear in daily life. Slattery tested the ability of human listeners to localize broadband noise bursts in the absence of binaural localization cues. The patients demonstrate that monaural cues can provide useful localization information in the horizontal as well as in the vertical dimension [2]. However, they often face barriers for speech communication in a noisy environment, especially when the sound source is on the bad ear side [3]. Patients with SSD often need to turn their head when communicating with others in order to use their good ears, with some patients feeling embarrassed or inconvenienced [4]. Moreover, the SSD patients are not able to distinguish the source of the sound.

Current intervention options for SSD include cochlear implants and hearing aids. Previous studies have shown that cochlear implants in SSD patients can improve speech recognition and sound source localization [5, 6]. However, studies have shown that SSD patients have different benefits after cochlear implantation, and cochlear implants are expensive, which makes patients unwilling to choose cochlear implants. As for hearing aids for SSD intervention, air conduction hearing aids and bone conduction hearing aids can be used. Air conduction hearing aids can be implemented on the bad side or on the healthy side by means of signal transmission. Contralateral routing of the signal system is a choice. Both ears need to be equipped with hearing aids. The auxiliary hearing device is worn on the bad ear to receive signals and transmit them to the contralateral ear. The main hearing device is worn on the good ear to receive and amplify the contralateral signals. Bone conduction hearing aid is another treatment method for SSD, which includes surgically implanted hearing aids and nonsurgically implanted hearing aids. However, implantable bone conduction hearing aids fix the sound processor onto the skull, which generates a large amount of pressure on the skull and can stimulate skin hyperplasia and cause pain in the patient. In addition, the transmission of sound is weakened due to the barrier of soft tissue. Nowadays, BAHA (Cochlear in Australia) is the most commonly used bone conduction hearing aid, which requires surgical implantation. Many patients have concerns about the impact of surgery and the infection on the wound. Studies have shown that 29% of surgical patients will experience infections near the implanted device, soft tissue proliferation, skin irritation, and displacement of implant [7]. In addition, bone conduction hearing aids offer no obvious hearing improvement at frequencies above 4 kHz [8]. Some studies [9–11] have shown that the average air conduction hearing threshold of 0.25 to 4 kHz after the subject wears BAHA was improved by 30.2 to 39.1 dB. Xia et al. tested [12] 12 cases wearing soft-band bone conduction hearing aids; the SRT was improved to 5.91 dB, which was better than naked ears with 13.64 dB. BAHA also has defects in the localization of the sound source in patients with extremely severe SSD. Currently, some controversies about hearing aid gain, sound source localization ability, and speech recognition under noise in implanted patients exist [13, 14]. Therefore, some patients with SSD are reluctant to use implantable bone conduction hearing aids [15, 16].

The advantage of nonimplantable bone conduction hearing aids is that they do not require complicated surgical procedures. A nonimplantable bone conduction device on the skull [17] requires placing a behind-the-ear (BTE) microphone in a hearing-impaired ear to simulate the acoustic characteristics of normal auricles. SoundBite bone conduction hearing aid is such a hearing device. The microphone receives the sound, and then, the sound is processed by BTE digital audio equipment. A removable in-the-mouth (ITM) device is fixed onto the teeth and directly coupled to the skull. The ITM device generates vibration that passes through the skull to the cochlea. The ITM device is directly fixed onto the dental bones, and the sound transmission will not be hindered by soft tissues. The sound transmission efficiency is higher than that achieved by adhesion or clamping.

A previous study compared eight bone conduction hearing aids' maximum output and gain; the researchers found that within the frequency range of 4 to 8 kHz, the maximum output and gain for each bone conduction device were different, with SoundBite demonstrating better performance [17]. In another study, the researchers measured the speech recognition threshold (SRT) with the noise from different directions while wearing SoundBite for SSD patients. The results showed that the SRT was an average of 2.5 dB lower than that without wearing SoundBite with the signal coming from the front and the noise from the good ear side. The SRT did not change when the noise came from the front. The SRT was reduced by 2.3 dB when the noise came from the bad ear side [18]. Also, studies have shown that SoundBite is comparable to, or even better than, BAHA in English speakers.

To date, however, there is no research investigating SoundBite bone conduction hearing aids in Chinese SSD patients. In this study, Chinese speech recognition in quiet and in noisy environments was evaluated before and after wearing the hearing aids. The GBI scale is designed for use only once postintervention, as a measure of change related to a specific surgical or medical intervention. The questionnaire consists of 18 questions answered using a five-point Likert scale, addressing change in health status post any intervention. The responses are then scaled and averaged to give a score with a range -100 (poorest outcome) through 0 (no change) to +100 (best outcome). A positive value indicates that the patient has benefited to a certain degree in quality of life after medical intervention, a zero score indicates no change, and a negative value indicates that the health level has deteriorated after the intervention. It is widely used in otolaryngology to report change in the quality of life postintervention [19]. The GBI scale was used to assess the impact of patients' general conditions, social support, and physical health benefit of hearing aids and to explore the clinical application of SoundBite bone conduction hearing aids in Chinese patients with SSD.

## 2. Materials and Methods

### 2.1. Ethics Statement

The study and the informed consent procedures were approved by the local ethics committee (Ethics Committee of the Shanghai Sixth People's Hospital, approval number: 2018-092), and written informed consent was obtained before participation.

### 2.2. Enrollment Indications

SSD patients who were 18 years or older were enrolled in this study. All the enrolled patients should have SSD history longer than 3 months. Before fitting a SoundBite appliance (Sonitus Medical Technology Company, Shanghai), the teeth of the patients were examined completely by a dentist to make sure that the teeth are healthy; there can be no active caries or periodontal or endodontic conditions affecting the abutment teeth [4].

Subjects were excluded if they met any of the following criteria: (1) currently using other hearing aids, such as BAHA, contralateral routing of signals, and TransEar; (2) speech recognition score (SRS) is disproportionately lower than what would be predicted with the pure tone audiometry (PTA); and (3) patients have psychological or mental conditions that may interfere with understanding, informed consent, compliance, and cooperation.

### 2.3. Basic Characteristics of Patients

Altogether, 9 patients with SSD for more than 3 months were enrolled in this study. The patients aged between 24 and 61 years with an average age of 39.3 ± 10.8 years, including 4 males and 5 females (see Table 1). The patients could understand and repeat Mandarin and demonstrated good compliance to wearing the hearing aid and to the evaluation of the hearing aid. The subjects had an average hearing threshold on the good ear ≤ 25 dB HL across 0.5, 1, 2, and 4 kHz. The etiology for SSD includes 5 cases of sudden deafness, 3 cases of postoperative acoustic neuroma, and 1 case of congenital unilateral sensorineural hearing loss.

### 2.4. Hearing Aid Fitting

During the whole study, the devices were fitted using the open SoundBite fitting software. The gain was adjusted to the most comfortable level for the patient. PTA was tested after adjustment. The fitted PTA must meet the criteria that the difference between the average air conduction hearing threshold (averaged across 0.5, 1, 2, and 4 kHz) after fitting and that in the healthy ear is within 15 dB. Feedback noise cancellation was turned on if there was howling.

### 2.5. Testing Procedure

The patients completed the PTA and tympanogram test before wearing a hearing aid. The Middle Ear Analyzer (Flute Basic, Italy) was used for the tympanogram. PTA tests were performed in a sound-proofed room with noise less than 30 dB (A) and a calibrated GSI-61™ audiometer (The United States) coupled with TDH 39 headphones.

The speech audiometry test was performed in a standard sound-proofed room with noise less than 30 dB (A) calibrated sound field. Before the test, the subjects were familiarized with the test process. The subjects were required to repeat what they heard; then, the audiologist judged whether the restatement was correct. After the test, the system will automatically calculate the SRS and display the results.

The materials named XinAiFeiYang issued by the People's Liberation Army General Hospital of Chinese were used for disyllable word recognition in quiet. The material includes 5 test lists, each list contains 40 words, which are enough to make the consonants and tones present in each list representative of those in the language used in daily life [20]. It has been clinically verified by many centers that it can meet the clinical requirements for test reliability, validity, and practicality [21]. Disyllable word recognition test was performed with a calibrated audiometer (Astera Conera, Denmark). TDH39 headphones were used to test the disyllable word SRS at 50 and 65 dB sound pressure level (SPL) which represents low and medium sound levels for communication.

The Mandarin HINT materials were used for SRS under noise. The Mandarin HINT test materials were donated by the House Ear Research Institute. It includes an exercise list, 12 test lists, and 20 sentences each list. Two calibrated loudspeakers (System 600, Tannoy) were used to present the sound. Both loudspeakers were placed at 1 m distance from the subject's head. The SRS under noise was evaluated under the following sound field conditions: (1) the signal sound came from the bad ear side and the noise from the good ear side (S_SSD_N_AH_), (2) the signal sound came from the front of the patient and the noise from the bad ear side (S_0_N_SSD_), and (3) the signal and noise both came from the front of the patients (S_0_N_0_). The noise for SRS is steady-state noise, which is spectrally matched to the average spectrum of the sentences. The sentence recognition score was measured at a noise intensity of 65 dB SPL and SNR of -2 and -5 dB.

The subjects underwent PTA, the abovementioned speech audiometry test before and after one month of wearing hearing aids, and the GBI questionnaire test one month after wearing a hearing aid. The impacts of SoundBite bone conduction hearing aids on hearing and speech audiometry results were analyzed before and after wearing the hearing aids, and the GBI questionnaire score result was analyzed after wearing the hearing aids.

All tests and the GBI questionnaire evaluation were performed by an experienced and professionally trained audiologist.

### 2.6. Statistical Analysis

Statistical analysis was carried out using the Statistical Package for Social Sciences (SPSS) version 19.0 (Chicago, IL, USA). The hearing thresholds, SRS under quiet and noisy environment between before and after wearing hearing aids, were compared with paired-sample *t*-tests.

## 3. Results

### 3.1. Pure Tone Audiometry

**Figure 1:**
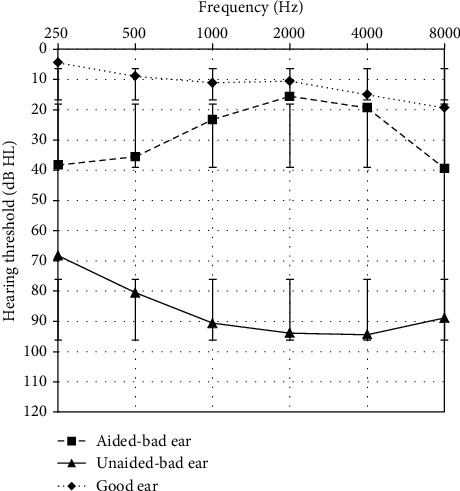
Average pure tone thresholds of SSD patients at frequencies of 250 to 8000 Hz before and after wearing a SoundBite bone conduction hearing aid. The lines represent the threshold of the good ear, the bad ear without the hearing aid, and the bad ear with the hearing aid. The bars represent one standard deviation.

The average hearing threshold of good and bad ears before wearing the hearing aids was 11.4 ± 2.6 dB HL and 89.9 ± 6.4 dB HL, respectively. The average hearing threshold of bad ears after fitting the hearing aid was 23.5 ± 9.0 dB HL (see Figure 1), representing a significantly improved hearing of 66.4 ± 14.9 dB compared to that before wearing the hearing aid (*p* < 0.001).

### 3.2. Speech Audiometry in Quiet

**Figure 2:**
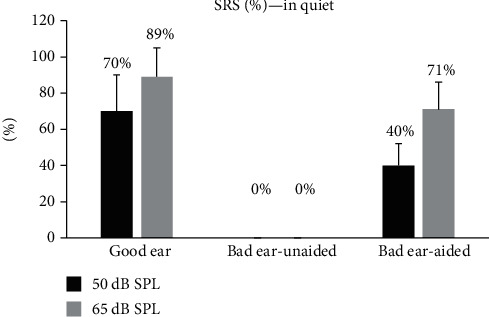
Disyllable word SRS under quiet environment of the good ear and the bad ear before and after wearing SoundBite bone conduction hearing aids for SSD patients at sound intensity 50 and 65 dB SPL. The bars represent one standard deviation.

The disyllable word SRS under quiet condition at 50 and 65 dB SPL for the good ears was 70 ± 20% and 89 ± 16%. The SRS in quiet at the two intensities was both 0% for the bad ears before wearing the hearing aids. After wearing the hearing aid, the SRS for the bad ears, obtained at 50 and 65 dB SPL, was increased by 40 ± 12 and 71 ± 15 percentage points (see Figure 2), respectively. The differences of SRS between before and after wearing the hearing aid were significant for both speech intensities (*p* < 0.001).

### 3.3. Speech Audiometry in Noisy

The speech recognition in noise was evaluated with sentence materials. When the signal came from the bad ear side and the noise came from the good ear side (S_SSD_N_AH_), the SRS scores were 28 ± 17% and 9 ± 10% without a hearing aid with the SNR at -2 dB and -5 dB, respectively. After wearing the hearing aid, the SRS scores were 45 ± 16% and 18 ± 9%, which significantly increased by 17 ± 6 and 9 ± 4 percentage points compared to that before wearing the hearing aid (*p* < 0.001).

When the signal came from the front of the patient and the noise came from the bad ear side (S_0_N_SSD_), the SRS scores were 95 ± 3% and 84 ± 7% without a hearing aid with the SNR at -2 dB and -5 dB, respectively. After wearing the hearing aid, the SRS scores were 90 ± 5% and 77 ± 8%. The SRS scores were reduced by 5 ± 5 and 7 ± 5 percentage points with the two SNRs, respectively. The differences between the two SRSs before and after fitting the hearing aid were statistically significant (*p* < 0.05).

**Figure 3:**
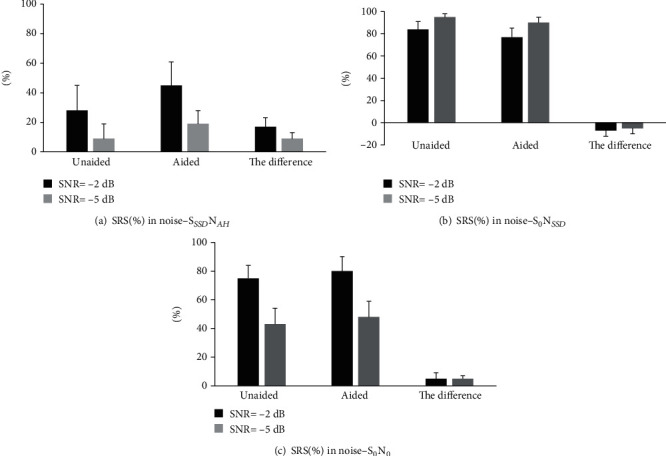
SRS under noisy environment before and after wearing SoundBite bone conduction hearing aids for SSD patients. The noise was at 65 dB SPL, and the SNR was equal to −2 and −5 dB. (a) S_SSD_N_AH_: the signal sound came from the bad ear side and the noise came from the good ear side. (b) S_0_N_SSD_: the signal sound came from the front of the patient and the noise came from the bad ear side. (c) S_0_N_0_: the signal and noise both came from the front of the patients. The bars represent one standard deviation.

When the signal and noise both came from the front of the patients (S_0_N_0_), the SRS scores were 75 ± 9% and 43 ± 11% without a hearing aid with the SNR at -2 and -5 dB, respectively. After wearing the hearing aid, the SRS scores were 80 ± 10% and 48 ± 11% with the two SNRs, respectively. The SRS was not significantly increased by5 ± 4 percentage points with the SNR at -2 dB compared with that before wearing hearing aids (*p* > 0.05); however, the SRS was significantly increased by 5 ± 2 percentage points with the SNR at -5 dB compared with that before wearing hearing aids (*p* < 0.05) (see Figure 3).

### 3.4. GBI Score

**Figure 4:**
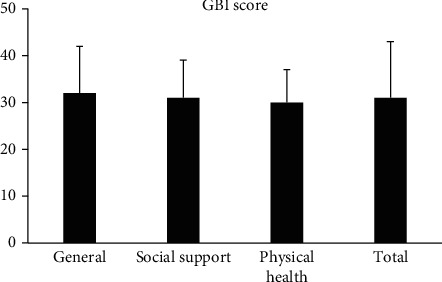
Glasgow Benefit Inventory (GBI) scores after wearing SoundBite bone conduction hearing aids for SSD patients. The total score and three subscale scores of general, social support, and physical health are shown separately. The bars represent one standard deviation.

The average GBI total score from the nine patients was 31 ± 12, with the average scores for the three subscales of general, social support, and physical health of 32 ± 10, 31 ± 8, and 30 ± 7, respectively. The results of the questionnaires showed that patients' quality of life improved significantly after wearing SoundBite bone conduction hearing aids (see Figure 4).

## 4. Discussion

In this study, 9 patients with SSD wore SoundBite bone conduction hearing aids for one month. Their hearing, speech recognition, and life benefits were evaluated before and after one month of wearing. This study was first performed in Mandarin Chinese speakers with speech tests using Chinese materials. The results showed that after wearing the hearing aid, the air conduction hearing threshold (across 0.5-4 kHz) decreased by 66.4 ± 14.9 dB for the bad ear. Under a quiet environment, the disyllable word SRS in the bad ear was improved by 40 ± 12 and 71 ± 15 percentage points after wearing the hearing aid with the speech signal at 50 and 65 dB SPL, respectively. Under a noisy environment, the SRS was increased by 17 ± 6 and 9 ± 4 percentage points with S_SSD_N_AH_ at SNR -2 and -5 dB, respectively. The SRS scores were reduced by 5 ± 5 and 7 ± 5 percentage points with S_0_N_SSD_ at SNR -2 and -5 dB, respectively. The SRS scores were improved by 5 ± 4 and 5 ± 2 percentage points with S_0_N_0_ at SNR -2 and -5 dB, respectively. The GBI benefit scale showed that the general conditions, social support, and physical health were improved after wearing the hearing aid.

Our study showed that the average aided hearing threshold for frequencies across 0.5, 1, 2, and 4 kHz was 23.5 dB HL. These results are consistent with previous studies, which showed the aided threshold of 21.3 dB for frequencies across 1, 2, 3, 4, and 6 kHz after wearing SoundBite for one month [22]. A previous study showed that BAHA is less effective at a frequency compensation of 4 kHz and above. This may be related to the attenuation of high-frequency sound vibration through subcutaneous tissues; thus, the acoustic signal could be weakened by 10-15 dB [23]. Our study showed that the average hearing threshold for frequencies across 4 and 8 kHz after wearing SoundBite was 29.4 dB HL. Therefore, SoundBite hearing aids are better than BAHA in the improvement of hearing at medium and high frequencies, which is critical for speech clearance and helps to improve speech intelligibility and speech recognition [14].

Disyllable word has an important value in auditory speech evaluation [24]. The results of this study showed that in patients with SSD, the SRS for disyllable word under quiet is increased with the signal at the bad ear side, especially at a moderate sound level. The SRS at the soft sound level of 50 dB SPL increased by 40 percentage points, and the SRS at a moderate sound level of 65 dB SPL increased by 71 percentage points after wearing the hearing aid, representing significant differences compared to that before wearing the hearing aid (*p* < 0.001). After wearing SoundBite, the improvement at moderate sound intensity was better than that at soft sound intensity, suggesting that the speech recognition of patients in a quiet environment was greatly improved at the everyday life sound level.

Spatial hearing and binaural hearing play an important role in the localization of sound sources, especially in a noisy environment. The main reason for speech recognition disturbance in a noisy environment for patients with SSD is the head shadow effect [25]. This is a physical phenomenon caused by the blocking of the sound by the head. When the sound reaches the opposite ear, it is attenuated and results in the SNR of the side closer to the signal higher than that in the other ear. Therefore, people are able to benefit from the head shadow effect regardless of the direction of the noise [26]. The results of this study showed that speech recognition improved significantly in both SNRs of -2 and -5 dB. In a previous study of 28 patients who wore SoundBite for 6 months, the speech recognition threshold decreased by 2.5 dB [18], which is equivalent to 25 percentage points increase in SRS, as 1 dB decrease in speech recognition threshold is equivalent to 10 percentage points increase in SRS [27]. The difference between these two studies could be explained by the difference in the configuration of speech and noise. Although the noise in both studies was on the good ear side, the voice in this study came from the bad ear side while the speech in Murray's study came from the front. The head shadow effect in this study increased the difficulty of speech recognition. Therefore, the SRS in noise seems to be a little lower than that in Murray's study. Moreover, the small sample size of this study and the short wearing time may also explain the difference between these two studies. A previous study comparing the SRT after wearing SoundBite one day and one month showed that the increase by 0.8 dB between one day and one month was statistically significant [18]. Therefore, the speech improvement could be more significant with the extension time of wearing the hearing aids. The speech audiometry was evaluated after one month of wearing in this study, while it was evaluated after 6 months of wearing in Murray's study. It may be expected that the effect on SRS will gradually increase with the longtime use of the hearing aid by the patients. The improved head shadow effect in SSD patients wearing SoundBite could be explained by the output of the device. Mark found that the maximum output frequency of SoundBite is above 2 kHz, which help the patients overcome the head shadow effect, so the SRS under noise was improved significantly by SoundBite bone conduction hearing aids [17].

When the signal and noise both came from the front of the patients (S_0_N_0_), both ears receive the signal and noise, which is different from listening with one ear, the subject hears louder. The subjects rely on the redundant information provided at the two ears, enhanced detection of smaller differences in signals, and improved speech recognition [28]. The auditory system is able to adjust the signals arriving at both ears by using the distinct time, level, and spectral cues occurring between the two ears. This permits a better separation of target and masker and improves intelligibility of the desired signal [29]. Gantz studied 10 patients with bilateral cochlear implants at condition S_0_N_0_ and found the SRS increased by 10.6 percentage points [30]. Our result obtained from the condition S_0_N_0_ showed that the SRS under noise environment increased significantly after wearing the hearing aid, which is consistent with the results obtained from bilateral cochlear implants.

While for the condition S_0_N_SSD_, the signal came from the front of the patient and the noise from the bad ear side, the noise and the signals are spatially separated. Our results show that the SRS deteriorates after wearing aid. The main reason is that when the noise is located at the bad ear side, the noise is amplified by the SoundBite and also be transmitted to the good ear side. As a result, the SNR of the good ear decreases and the noise interference results in a decrease in speech recognition.

SSD patients cannot accurately determine the sound source and need to turn their heads to find the sound. Moreover, speech recognition is not ideal in a noisy environment, which greatly affects the quality of life of patients [31, 32]. The abbreviated profile of hearing aid benefit (APHAB) hearing aid gain scale is usually used to evaluate the benefit of SoundBite. Studies have shown that the average APHAB score with SoundBite was 23.2, which was higher than the BAHA score -7 to 17 [33–36]. In this study, the GBI benefit scale was used to evaluate the general conditions, social support, and physical health of patients with SSD after wearing SoundBite. The average score after wearing SoundBite was 31 ± 12. A previous study using GBI to evaluate the life quality showed that GBI questionnaire total score with the Sophono Alpha 2 transcutaneous bone-anchored sound processor was 14 ± 11.0, with subscale general situation score 18 ± 18.3, social support score 18 ± 22.7, and physical health score −4 ± 11.1 [37]. The scores in all aspects were worse than those of the SoundBite in this study, especially for the impact of hearing aids on physical health. The main reason for significant improvement is that the SoundBite does not require surgery, which reduces the patient's fear and the chance of postoperative infection. Also, the device without surgery can gain more support from family members and friends. The easy procedure to remove the device also makes the subjects feel convenient. All these factors result in higher GBI score compared with that of implanted bone conduction hearing aids.

## 5. Conclusions

SoundBite bone conduction hearing aids are beneficial for SSD patients. It could improve the speech recognition ability of patients in a quiet and noisy environment and quality of life after wearing it for one month. However, the sample size in this experiment is small, and the long-term effects of this device on the speech recognition and quality of life under various listening environment should be explored in further clinical research.

## Figures and Tables

**Table 1:** Characteristics of the single-side deafness patients in this study.

Subject no.	Gender	Age	Deafness ear	Duration of deafness	Causes of deafness
01	Male	61	Right	20 years	Sudden deafness
02	Male	35	Right	20 years	Postoperative acoustic neuroma
03	Female	41	Left	2 years	Postoperative acoustic neuroma
04	Male	40	Right	2 years	Sudden deafness
05	Female	24	Right	3 years	Sudden deafness
06	Female	30	Right	2 years	Sudden deafness
07	Male	46	Left	6 months	Sudden deafness
08	Female	35	Left	More than 30 years	Congenital deafness
09	Female	44	Right	7 years	Postoperative acoustic neuroma

## Data Availability

The form data used to support the findings of this study are available on request to the corresponding author: Dr. Yanmei Feng, email: feng.yanmei@126.com.

## Additional Points

Research direction is audiology and vestibular function evaluation.
